# Factors Influencing Patient-Centeredness among Korean Nursing Students: Empathy and Communication Self-Efficacy

**DOI:** 10.3390/healthcare9060727

**Published:** 2021-06-12

**Authors:** Jaehee Jeon, Seunghye Choi

**Affiliations:** 1 Department of Nursing, Gangneung-Wonju National University, Wonju-si 26403, Korea; anesjjh@naver.com; 2 College of Nursing, Gachon University, Incheon 21936, Korea

**Keywords:** nursing student, patient-centered, empathy, communication, self-efficacy

## Abstract

In recent years, there is a growing tendency in the extent to which patients wish to be actively involved in processes related to their healthcare and relevant decision-making. This was a cross sectional study. We examined undergraduate nursing students’ patient-centeredness and investigated its associated factors including empathy and communication self-efficacy using a structured questionnaire. A total of 201 undergraduate nursing students who provided written consent to participate in the study completed measures on patient-centeredness (sharing and caring), empathy (fantasy, perspective taking, personal distress, and empathic concern), and communication self-efficacy. The factors affecting patient-centeredness were analyzed using multiple regression. Communication self-efficacy affected patient-centered sharing, while age, empathy (fantasy, personal distress, empathic concern), and communication self-efficacy affected patient-centered caring. Empathy and communication self-efficacy positively affected patient-centeredness. Therefore, strategies that promote empathy and communication self-efficacy are needed to increase patient-centered care competency.

## 1. Introduction

In recent years, there is a growing tendency in the extent to which patients wish to be actively involved in processes related to their healthcare and relevant decision-making [1]. The World Health Organization states that healthcare recipients’ responses should be included in healthcare goals and that healthcare services must meet consumers’ needs and expectations [2]. To keep abreast with these changes, person-centered care, also known as patient-centered care or client-centered care, is gaining traction in the field of nursing as well [3]. However, ontological assumptions of patient-centered care are diverse [4]. According to previous studies, patient-centered care consists of patient perceptions of the degree to which key communication outcomes (e.g., information exchange, managing uncertainty, etc.) were achieved [5]. Moreover, patient-centered care consists of patient perceptions of the quality of clinician communication with respect to information-giving, decision-making, building relationships, managing uncertainty, and responding to emotions, according to another study [6]. Although there are various assumptions about patient-centered care, most previous studies treat communication as a major component of it.

Another factor that could contribute to patient-centered care is empathy [7]. The goal of communication to implement patient-centered care is not just information exchange, but also building relationships [6]; therefore, empathy is important. Empathy, respect for individuality, communication, and comfort have been identified as some competencies required for nurses to provide patient-centered care [8]. Therapeutic communication refers to a nursing skill that allows the patient to express his/her current situation, goals, emotions, and health problems related to his/her treatment/recovery to the nurse, and the nurse facilitates interaction with the patient in order to resolve problems and meet the nursing goals [9]; this competency can be improved through continuous experience with interpersonal relationships. On the other hand, empathy involves nonverbal skills, such as listening, contact, and physical movement, and verbal skills, such as reflection and paraphrasing, and is based on respect and consideration for the other person; although it is difficult to acquire this skill in the short term, it was reported that students could develop empathy with repeated practice [10].

Nursing students who will become nurses should establish effective interactions with patients to deliver patient-centered care [11,12]. Educational programs in colleges are crucial to equip nurses with competencies for patient-centered care [13]; however, research on nursing students’ patient-centeredness is not only scarce but also inconsistent. One study reported that because students tend to only focus on accurately performing nursing techniques, they do not pay attention to patients’ needs [14]. This may be due to nursing students spending limited time with patients during clinical practice [15]. However, another study reported that nursing students had higher patient-centeredness scores than nurses [16]. This result may indicate that the actual medical setting is not very conducive to the development of a patient-centered orientation and communication skills [17]. It is important to promote patient-centeredness competency in nursing students [12].

Therefore, this study aimed to examine the level of patient-centeredness among Korean undergraduate nursing students and investigated the factors that affect it, including empathy and communication self-efficacy.

## 2. Methods

### 2.1. Sample and Procedure

This was a cross-sectional survey. Undergraduate nursing students from a single nursing school in Korea who were at least 19 years of age and provided informed consent were enrolled in this study. Students already participating in empathy or communication education as an extracurricular activity outside of nursing school were excluded.

The sample size was estimated using G*Power 3.1.9.2 [18]. The total sample size required was 176 for a *t*-test, 84 for ANOVA, and 53 for multiple regression, for a medium effect size (0.25), α = 0.05, and power (1-β) = 0.95. Considering potential withdrawals, we recruited 202 participants. Data were collected between 1–28 September 2019, in a nursing school in South Korea. For participant recruitment, we obtained permission and cooperation from the dean of nursing at the school and posted a recruitment advertisement on the internet and departmental bulletin board. A research assistant explained the details of the study to nursing students who voluntarily expressed their willingness to participate in the study, and written informed consent was obtained prior to beginning the study.

### 2.2. Ethical Considerations

Before the study was conducted, the research proposal and questionnaire were approved by the Institutional Review Board of Gachon University (No. 1044396-201905-HR-070-01). All individuals gave their consent for test result notification and signed a personal information usage agreement. We provided a gift card to participants who completed the survey.

### 2.3. Questionnaire

A structured questionnaire was used for this study. The questionnaire consisted of 7 items on general characteristics (gender, age, school year, history of hospitalization or family’s hospitalization, satisfaction with major, satisfaction with training), the Patient-Practitioner Orientation Scale (PPOS; 18 items) to assess patient-centeredness, the Interpersonal Reactivity Index (IRI; 28 items) to assess empathy, and the Communication Self-Efficacy Scale (37 items).

The PPOS was developed to measure attitudes toward the practitioner–patient relationship, and we used the Korean version of the tool, which was modified and adapted for use with nurses and nursing students [16,19] based on the Korean tool validated for use with health professionals [20]. The PPOS comprises the sharing and caring subscales to identify whether the patient–practitioner relationship is more patient-centered or practitioner-centered [21]. The sharing subscale assesses the belief that healthcare professionals and patients have equal rights and decision-making power, and that healthcare professionals must share as much information as possible with patients. The caring subscale assesses the belief that exchange of emotions and a good relationship are key values and that healthcare professionals must provide holistic care for patients. Each item is rated on a 6-point Likert scale (1 = *absolutely agree* and 6 = *absolutely disagree*). A score closer to 6 indicates a patient-centered attitude, while a score closer to 1 indicates a practitioner- or disease-centered attitude. As for the reliability of the instrument, Cronbach’s α was 0.65 when used with nurses [19], 0.77 for the whole scale, 0.59 for the sharing subscale, and 0.70 for the caring subscale in this study.

Empathy was assessed using the IRI, originally developed by Davis [22], translated into Korean, and validated [23]. This 28-item instrument assesses empathy multi-dimensionally, with 7 items in each of the four subscales: perspective taking (PT), fantasy (FA), empathic concern (EC), and personal distress (PD) [23]. Negative items are reverse-coded. PT refers to the tendency to voluntarily adopt another person’s psychological perspective and attitude, and FA refers to the tendency to imagine oneself with the feelings and actions of fictitious characters in books, movies, and plays. EC refers to the tendency to feel other-oriented sympathy and concern for unfortunate others, and PD refers to the tendency to feel uneasy and distressed when witnessing others’ misfortune or pain [23]. Each item is rated on a 5-point scale (1 = *Does not describe me well* and 5 = *Describes me very well*). The total score for each item ranges from 7 to 35, and a higher score indicates a higher level of empathy in each subscale [23]. Cronbach’s α for the subscales ranged from 0.70 to 0.78 at the time of development [22] and was 0.67 for PT, 0.71 for FA, 0.65 for EC, and 0.73 for PD in the present study.

The Communication Self-Efficacy Scale was developed to be used with psychotherapists [24] and was modified by Park [25] for use with nursing students. This tool consists of 37 items (16 negative, 21 positive items) in 5 subscales, with 12 items on specific communication skills, 10 items on the counseling process, 7 items on handling difficult patient behaviors, 4 items on the ability to deal with cultural differences, and 4 items on the recognition of values. Each item is rated on a 6-point Likert scale (1 = *strongly disagree* and 6 = *strongly agree*). The score ranges from 37 to 222, with a higher score indicating a higher level of communication self-efficacy. Cronbach’s α was 0.88 at the time of development [24] and 0.81 in this study.

### 2.4. Statistical Analysis

The data were analyzed using SPSS version 25.0 (IBM Corp., Armonk, NY, USA). Of the 202 questionnaires, 1 was excluded for having missing data on more than one-third of the questions, and a total of 201 questionnaires were thus included in the analysis.

Sociodemographic characteristics and IRI, communication self-efficacy, and PPOS scores were summarized using frequencies, percentages, means, and standard deviations. The Shapiro–Wilk test was used to test the normality of the variables, and the test result was assumed to follow a normal distribution with a significance of *p* > 0.05. Differences in PPOS scores according to general characteristics were examined with *t*-test, ANOVA, and Scheffé post-hoc test for the sharing and caring subscales of the PPOS. The associations between IRI, communication self-efficacy, and PPOS scores were analyzed with Pearson’s correlation coefficient. The predictors of PPOS sharing and caring were analyzed using multiple regression. Among the general characteristics that had significant associations with patient-centered caring, school year was treated as a dummy variable for the multiple regression.

## 3. Results

### 3.1. Sociodemographic Characteristics

A total of 79.1% of the participants were women. The mean age was 21.53 years, and 57.2% of the participants were 22 years or younger. School year was evenly distributed, and no religion or other religion was the most common (37.1%), followed by Christian, Buddhist, and Catholic religions. A total of 84.1% reported a history of hospitalization or family’s hospitalization. More than 71.6% of the participants reported being satisfied/very satisfied with their major. Regarding satisfaction with clinical training, more than 25.4% reported being satisfied or very satisfied, 18.4% slightly dissatisfied, and 6.5% dissatisfied (Table 1).

### 3.2. Empathy, Communication Self-Efficacy, and Patient-Centeredness Scores

The empathy scores (IRI) were the highest for EC (26.45), followed by PT (26.00), FA (25.05), and PD (19.85); the mean communication self-efficacy score was 147.81; and the mean PPOS score was 4.18 for the sharing subscale and 4.26 for the caring subscale (Table 2).

### 3.3. Differences in Patient-Centeredness According to General Characteristics

Regarding the differences in patient-centeredness scores according to general characteristics, sharing scores differed significantly according to age (*F* = 3.10, *p* = 0.047), but post-hoc tests were not significant. Caring scores differed significantly according to age (*F* = 9.45, *p* < 0.001) and school year (*F* = 13.02, *p* < 0.001). Post-hoc tests revealed that caring scores were higher among those aged 22 years or older than those below 22 years of age, and higher among second-, third-, and fourth-year students than among first-year students (Table 1).

### 3.4. Correlations between Empathy, Communication Self-Efficacy, and Patient-Centeredness

The patient-centered sharing score was positively correlated with PD (*r* = 0.163, *p* = 0.021) and communication self-efficacy (*r* = 0.386, *p* < 0.001), and the caring score was positively correlated with FA (*r* = 0.298, *p* < 0.001), PD (*r* = 0.377, *p* = < 0.001), EC (*r* = 0.178, *p* = 0.012), and communication self-efficacy (*r* = 0.359, *p* < 0.001) (Table 3).

### 3.5. Factors Associated with Patient-Centeredness

Multiple logistic regression was performed to identify the factors that affect the sharing and caring components of patient-centeredness. The assumptions of regression were all met. Autocorrelation was tested with the Durbin–Watson statistic; the value for regression for the effect on the sharing component was 1.876, and that for caring was 1.883, which confirms the absence of autocorrelation. Next, we checked for multicollinearity using tolerance and variance inflation factor (VIF). As tolerance was 0.1 or greater, and VIF was less than 10, there was no problem of collinearity with any of variables. Each of the two regression models (sharing and caring) was found to be significant (*F* = 12.22, *p* < 0.001; *F* = 14.65, *p* < 0.001, respectively). First, communication self-efficacy (β = 0.36) had a significant effect on sharing, and the adjusted coefficient of determination (Adj R^2^) was 0.15 (Table 4). Next, communication self-efficacy (β = 0.27) had the greatest significant effect on caring, followed by FA (β = 0.23), PD (β = 0.17), age (β = 0.17), and EC (β = 0.16), with Adj R^2^ being 0.29 (Table 5).

## 4. Discussion

This study aimed to investigate patient-centeredness in undergraduate nursing students and identify the influence of empathy and communication self-efficacy.

In this study, the mean PPOS sharing subscale score was 4.18 ± 0.49, and the mean caring subscale score was 4.26 ± 0.51, which are lower than the values found in a previous study with undergraduate nursing students (sharing 4.43 ± 0.66 and caring 4.71 ± 0.49) [16]. In a previous study comparing undergraduate nursing students and nurses, the caring subscale score was similar in the two groups, but the sharing subscale score was higher in the undergraduate nursing students than in nurses (4.43 vs. 4.25). The reason for this has been reported to be that current nursing curricula emphasize communication and patients’ disease experience [16]. In our study, the caring subscale score was significantly higher among second-, third-, and fourth-year students than among first-year students (*p* < 0.001), but sharing subscale scores did not differ significantly according to school year. The PPOS sharing subscale includes the belief that healthcare professionals and patients have equal rights and decision-making power, and that healthcare professionals must share as much as information as possible with patients. Such belief has been reported to be affected by cultural factors such as a hierarchical, paternalistic culture among healthcare professions [26]. Traditionally, physicians have stronger rights and decision-making power in Korean culture [27]. However, patients’ rights are becoming increasingly important over time, and hospital culture is shifting toward one that respects patients’ opinions [28]. Because our study participants were from a single region, studies with nursing students from several regions are needed to examine whether there are regional differences in the degree of sharing according to cultural background. Moreover, nurses’ patient-centeredness was found to be associated with patient’s self-care ability in the future [29]. A previous study reported that students mainly focus on accomplishing procedures and tasks correctly, rather than patients’ need and worries [30]. Therefore, nursing educational strategies that enhance patient-centered sharing and caring are needed. In previous studies with medical students, the PPOS score was higher for women than for men [31]; however, there were no significant gender differences in our sample of Korean undergraduate nursing students. This is consistent with the results of previous studies among nursing students [16]. According to a previous study, when the level of empathic responses was assessed, no differences between male and female students was found; however, female students had more complex maps that included a larger number and levels of empathy-related concepts [32]. Although there were no significant differences, the mean value of PPOS score was higher in women than men in our results. Therefore, this study will need to be replicated with larger samples.

In the regression analysis, communication self-efficacy had a significant impact on the sharing scores, while communication self-efficacy, age, and the FA, PD, and EC subscales of the IRI had an impact on the caring scores. Communication self-efficacy affected both sharing and caring aspects of patient-centeredness. According to a previous study, patient-centeredness involves respecting patients’ preferences, needs, and values and considering these in decision-making; thus, nurses’ ability to empathize with patients and accurately communicate with them has been reported to be essential [33]. Moreover, patient-centered communication is a core competency in modern healthcare and is associated with improved patient satisfaction and health outcomes, as well as lower levels of burnout among medical staff [34]. Although we did not directly measure nursing students’ communication abilities in this study, considering that self-efficacy is defined as confidence or beliefs in one’s capabilities to organize and execute the courses of action required to produce given attainments [35], and that the scale used in this study assesses communication ability multi-dimensionally, the scores might indirectly reflect the actual level of communication competency among participants. In our study, communication self-efficacy was a positive predictor of both aspects of patient-centeredness, highlighting the need for curricula that emphasize nursing students’ patient-centered communication. A prior study found that the educational curriculum affects patient-centered attitudes [36]. In South Korea, a 5-year accreditation cycle by the Korean Accreditation Board of Nursing Education (KABOBE) is conducted to ensure quality in all baccalaureate nursing programs [37]. To qualify the criteria of KABOBE, many schools are making efforts to help students promote humanistic values and perspectives [37]. Nevertheless, few studies regarding person-centered care competency exist among Korean nursing students [37]. According to a previous study, there are three parts in a curriculum: a formal curriculum outlines what is planned to be taught (and how), the informal curriculum refers to what is actually taught (including unscripted teaching), and the hidden curriculum refers to ‘what is being experienced’ by the students (including information implicitly conveyed by teachers and peers and the values and moral judgements of the profession) [38]. Thus, structuring non-curricular activities to improve patient-centeredness would also be needed.

According to Rogers [39], empathy is not simply a communication skill but the basic attitude of empathizing. Furthermore, one needs to be equipped with the ability to understand others’ perspectives or stance in order to have an empathetic interest in others beyond oneself, and such ability can be acquired through education [40]. Empathy is typically seen as a multidimensional construct that includes cognitive and affective components [22]. Indeed, the IRI consists of four subscales grouped into cognitive empathy (PT, FA) and affective empathy (EC, PD) [22]. In this study, no empathy components significantly affected patient-centered sharing, while the FA, PD, and EC components of empathy were positive predictors of patient-centered caring. The EC and PC components (affective empathy) are involved in the mediating stage between the patient and care provider. EC is the factor that prompts one to act for the welfare of the other, and PD refers to the process of mediation to have the other person move away from pain. Cognitive empathy refers to intellectually taking the role or perspective of another person and seeing the world as they do [22]. In our study, empathy was a significant predictor of caring. Moreover, empathy training was found to be effective [41]. Therefore, educational strategies should be developed to enhance nursing students’ empathy.

This study has some limitations. As the data were collected from a single university in Korea, the findings cannot be generalized to the entire Korean nursing student population. Further, unlike previously reported, nursing students’ patient-centeredness did not significantly differ according to gender, and this may be due to the lower percentage of male students in our study population. In the future, multicenter studies are needed to develop nursing programs that could improve communication efficacy and empathy competency, which were found to affect nursing students’ patient-centeredness.

## 5. Conclusions

This study found that empathy and communication self-efficacy have an effect on nursing undergraduates’ patient-centeredness. In particular, communication self-efficacy had an impact on patient-centered sharing and caring. However, because sharing did not increase with school year, it is important to educate Korean nursing students about the need to share care-related information with patients as much as possible. Age, empathy, and communication self-efficacy were positive predictors of the caring aspect of patient-centeredness. Thus, it is important to develop strategies to improve nursing students’ empathy and communication abilities through curricular and non-curricular programs in order to enhance their patient-centeredness.

## Figures and Tables

**Table 1:** Participant Characteristics and Related Differences in Patient-Centeredness as Measured by the Patient–Practitioner Orientation (PPOS) Scale (*n* = 201).

Characteristics	Categories	*n* (%)	Sharing Subscale (PPOS)		Caring Subscale (PPOS)	
			Mean ± SD	*t* or *F* (*p*) Scheffé	Mean ± SD	*t* or *F* (*p*) Scheffé
Gender	Male	42 (20.9)	4.08 ± 0.53	−1.53 (0.127)	4.26 ± 0.46	0.11 (0.918)
	Female	159 (79.1)	4.21 ± 0.47		4.26 ± 0.53	
Age (years)	<22 ^a^	115 (57.2)	4.11 ± 0.45	3.10 (0.047)	4.13 ± 0.48	9.45 (<0.001) a < b,c
	22 to <25 ^b^	72 (35.8)	4.23 ± 0.53		4.42 ± 0.55	
	≥25 ^c^	14 (7.0)	4.32 ± 0.47		4.51 ± 0.27	
	Mean ± SD	21.53 ± 2.35				
Grades	First ^a^	50 (24.9)	4.03 ± 0.52	2.21 (0.088)	3.93 ± 0.46	13.02 (<0.001) a < b,c,d
	Second ^b^	50 (24.9)	4.19 ± 0.42		4.22 ± 0.46	
	Third ^c^	51 (25.4)	4.24 ± 0.44		4.40 ± 0.49	
	Fourth ^d^	50 (24.9)	4.25 ± 0.54		4.47 ± 0.48	
Hospitalization of oneself or family	Yes	169 (84.1)	4.18 ± 0.48	0.06 (0.950)	4.26 ± 0.52	−0.12 (0.904)
	No	32 (15.9)	4.17 ± 0.54		4.27 ± 0.48	
Satisfaction with major	Very satisfied	39 (19.4)	4.30 ± 0.50	1.17 (0.323)	4.22 ± 0.49	1.64 (0.182)
	Satisfied	105 (52.2)	4.16 ± 0.50		4.33 ± 0.56	
	Slightly dissatisfied	48 (23.9)	4.11 ± 0.44		4.13 ± 0.39	
	Dissatisfied	9 (4.5)	4.20 ± 0.47		4.22 ± 0.62	
Satisfaction with clinical practice	Very satisfied	6 (3.0)	4.09 ± 0.32	0.79 (0.500)	4.17 ± 0.44	0.64 (0.589)
	Satisfied	45 (22.4)	4.29 ± 0.52		4.45 ± 0.46	
	Slightly dissatisfied	37 (18.4)	4.27 ± 0.49		4.45 ± 0.50	
	Dissatisfied	13 (6.5)	4.09 ± 0.47		4.46 ± 0.55	

PPOS: Patient–Practitioner Orientation, SD: Standard Deviation.

**Table 2:** Scores on Interpersonal Reactivity Index, Communication Self-Efficacy, Patient–Practitioner Orientation Scale (*n* = 201).

Scale items	Number of Items	Possible Score Range	Mean ± SD	Mean ± SD/ Number of Items
**Interpersonal Reactivity Index**				
Fantasy (FA)	7	7–35	25.05 ± 3.84	
Perspective taking (PT)	7	7–35	26.00 ± 2.98	
Personal distress (PD)	7	7–35	19.85 ± 4.07	
Empathic concern (EC)	7	7–35	26.45 ± 3.28	
**Communication Self-Efficacy Scale**	37	37–222	147.81 ± 12.70	
**Patient–Practitioner Orientation Scale**				
Patient-centered Sharing	9	9–54	37.61 ± 4.37	4.18 ± 0.49
Patient-centered Caring	9	9–54	38.32 ± 4.63	4.26 ± 0.51

SD: Standard Deviation.

**Table 3:** Correlations between Interpersonal Reactivity Index, Communication Self-Efficacy, Patient–Practitioner Orientation Scale (*n* = 201).

Variables	Categories	Interpersonal Reactivity Index				Communication Self-Efficacy Scale	Patient–Practitioner Orientation Scale	
		FA	PT	PD	EC		Sharing	Caring
		*r* (*p*)						
**Interpersonal Reactivity Index**								
FA		1						
PT		0.173 (0.014)	1					
PD		0.260 (<0.001)	−0.185 (0.009)	1				
EC		0.410 (<0.001)	0.511 (<0.001)	0.083 (0.240)	1			
**Communication Self-Efficacy Scale**		0.130 (0.066)	0.292 (<0.001)	−0.327 (<0.0001)	0.286 (<0.001)	1		
**Patient–Practitioner Orientation Scale**								
Patient-centered Sharing		0.059 (0.403)	0.021 (0.764)	0.163 (0.021)	0.056 (0.434)	0.386 (<0.001)	1	
Patient-centered Caring		0.298 (<0.001)	0.008 (0.913)	0.377 (<0.001)	0.178 (0.012)	0.359 (<0.001)	0.486 (<0.001)	1

FA: Fantasy, PT: Perspective Taking, PD: Personal Distress, EC: Empathic Concern.

**Table 4:** Multiple Regression for Patient-Centered Sharing (Patient–Practitioner Orientation Subscale) (*n* = 201).

Variables	*B*	*SE*	β	*t*	*p*	Adj R^2^	*F* (*p*)
Constant	4.09	0.48		6.76	<0.001	0.15	12.22 (<0.001)
Age (years)	0.01	0.01	0.06	0.87	0.124		
Personal distress (empathy)	0.01	0.01	0.07	1.08	0.282		
Communication self-efficacy	0.01	0.03	0.36	5.37	<0.001		

Adj R^2^: Adjusted Coefficient of Determination, SE: Standard Error.

**Table 5:** Multiple Regression for Patient-Centered Caring (Patient–Practitioner Orientation Subscale) (*n* = 201).

Variables	*B*	*SE*	β	*t*	*p*	Adj R^2^	*F* (*p*)
Constant	1.71	0.62		2.75	0.007	0.29	14.65 (<0.001)
Age (years)	0.37	0.02	0.17	2.54	0.012		
Grades (fourth) *	0.13	0.08	0.11	1.56	0.121		
Fantasy (empathy)	0.03	0.01	0.23	3.18	0.002		
Personal distress (empathy)	0.02	0.01	0.17	2.42	0.016		
Empathic concern (empathy)	0.02	0.01	0.16	2.20	0.029		
Communication self-efficacy	0.01	0.03	0.27	4.35	<0.001		

*: Dummy Variable, SE: Standard Error.

## Data Availability

The data presented in this study are available on request from the corresponding author. The data are not publicly available due to protect the participants.
